# Serum Allergen-Specific IgE among Pediatric Patients with Primary Immunodeficiency

**DOI:** 10.3390/children9040466

**Published:** 2022-03-25

**Authors:** Karolina Pieniawska-Śmiech, Aleksandra Lewandowicz-Uszyńska, Magdalena Zemelka-Wiacek, Marek Jutel

**Affiliations:** 1 Department of Clinical Immunology, Wroclaw Medical University, 50-368 Wroclaw, Poland; magdalena.zemelka-wiacek@umw.edu.pl; 2 Department of Clinical Immunology and Paediatrics, Provincial Hospital J. Gromkowski, 51-149 Wroclaw, Poland; aleksandra.lewandowicz-uszynska@umw.edu.pl; 3 Third Department and Clinic of Paediatrics, Immunology and Rheumatology of Developmental Age, Wroclaw Medical University, 50-367 Wroclaw, Poland; 4 ALL-MED Medical Research Institute, 53-201 Wroclaw, Poland

**Keywords:** allergy, inborn errors of immunity, immunoglobulin E (IgE), primary immunodeficiency (PID), specific IgE (sIgE)

## Abstract

Background: Allergy is a clinical condition that reflects a deviated function of the immune system. The purpose of this study was to evaluate serum allergen-specific IgE (sIgE) along with clinical manifestations of allergy in patients with diagnosed primary immunodeficiency (PID). Methods: 72 patients, aged 1–17 years, diagnosed with PID and hospitalized between July 2020 and February 2021 were included in the study. Blood samples were obtained by venipuncture. sIgE (30 allergens), blood eosinophil count, as well as total IgE and IgG were measured and assessed in relation to a detailed medical examination. Results: Serum sIgE was detected in the blood of 50% of the patients in the study group, which significantly correlated (*p* < 0.0001) with clinical symptoms of allergy. During the period of the study, 61.1% of the patients showed symptoms of allergy, with 77.27% of them having tested positive for sIgE. The total IgE level was elevated in 18.06% of the patients and correlated with clinical symptoms of allergy (*p* = 0.004). An elevated total IgE level was not observed in children receiving immunoglobulin replacement therapy. Conclusion: The study showed that serum sIgE and total IgE together might be a plausible diagnostic tool for PID patients. However, for patients receiving immunoglobulin replacement therapy, the assessment of total IgE is not useful.

## 1. Introduction

Inborn errors of immunity (IEI) are a heterogeneous group of inherited diseases associated with genetic susceptibility, often stemming from a single-gene mutation. In the past, their prevalence was estimated at approximately 1:10,000–1:50,000. Due to novel diagnostic procedures, new nosologic units have been described and classified as primary immunodeficiencies (PID). The description of clinical phenotypes is being continuously updated [1,2]. Currently, the prevalence of IEI is estimated at 1:1000–1:5000 [3]. More than 400 diseases have been classified as PID. In most cases, they result from a single-gene mutation causing impaired development and function of the immune system. Regardless of their exact prevalence, PIDs are regarded as an unprecedented model, connecting defined monogenic defects with clinical manifestations of incorrect immune regulation. Immune dysregulation is a term used to characterize an array of autoimmune and inflammatory conditions [4]. In practice, IEI are associated with increased susceptibility to infection (especially severe, atypical, and/or recurrent infections), autoimmunity, allergy, and malignancy [3,5]. Immune dysregulation seems to be as important as susceptibility to infection in defining inborn errors of immunity nowadays. The International Union of Immunodeficiency Societies (IUIS) regularly provides an overview of IEI. In current IUIS classification, there are 10 IEI categories according to their underlying molecular defect, and one of them is called ‘diseases of immune dysregulation’. Within these IUIS classifications, each disorder is only listed once; therefore, these overviews do not feature phenotypic overlaps. However, it has been shown that other patients classified as having B-cell, T-cell, or innate immune system deficiencies are also at risk of autoimmune or inflammatory conditions [6].

Allergy develops due to a deviated function of the immune system. The occurrence of allergy in the context of PID has always been a compelling issue. Several PIDs are frequently associated with allergies. Moreover, common allergic reactions (eczema, allergic rhinitis, asthma, and food allergies) are exaggerated immune responses that may be manifestations of an underlying PID [7]. Appreciation of allergy as a manifestation of immune dysregulation should also stimulate the involvement of allergy specialists [8]. The misdiagnosis of PID as allergy has been described and may be complicated by the comorbidity of allergic diseases [8].

Atopic dermatitis (AD), in association with an elevated level of total IgE, constitutes a clinical feature of the hyper-IgE syndrome, Omenn syndrome, and Netherton syndrome. In the Wiskott–Aldrich syndrome (WAS), features of eczema are indistinguishable from those of atopic eczema. Selective IgA deficiency (sIgAD) often coexists with asthma, allergic rhinitis (AR), AD, and food allergy (FA) [9,10]. Allergic diseases, including food and drug allergies, are also present in other types of PID [11].

Studies on the prevalence of allergy in several PID disorders have shown mixed results. The reported differences might be due to differences in methodological approaches, as well as to ethnic and geographical diversity [12,13]. Thus, data on the prevalence of FA in patients with PID are very limited. The purpose of this study was to evaluate the prevalence of clinical manifestations of allergy and serum allergic-specific IgE (sIgE) in patients with PID.

## 2. Materials and Methods

The study was consented by the Bioethical Commission of the Wroclaw Medical University. Patients included in the study were previously diagnosed with PID (according to IUIS criteria and classification and ICD-10) and hospitalized in the Clinical Immunology and Pediatric Ward of the J. Gromkowski Provincial Hospital in Wroclaw from July 2020 to February 2021. All parents/legal guardians and patients over 16 years signed an informed consent before inclusion in the study.

The study group included 72 subjects (*n* = 46 boys, *n* = 26 girls) aged 1–17 years (median age = 7; mean age = 7.7). The baseline characteristics of the patients are presented in Table S1. Patients with predominantly antibody deficiencies (PAD; *n* = 51; 70.83%) constituted most of the study group, followed by patients with combined immunodeficiencies associated with syndromic features (*n* = 11; 15.28%). In addition, the study group was divided into two groups depending on the treatment received: a group of patients received permanent immunoglobulin (Ig) substitution therapy (*n* = 19; 26.39%), and a group did not receive Ig substitution therapy (*n* = 53; 73.61%) at the time of the study. The group of patients receiving Ig replacement therapy (IRT) included patients with X-linked agammaglobulinemia (*n* = 1), common variable immunodeficiency (CVID; *n* = 3), IgG subclass deficiency (*n* = 1), four patients with other hypogammaglobulinemias such as IgG deficiency (*n* = 3) and IgG subclass plus IgM deficiency (*n* = 1), one patient with Kabuki syndrome plus IgG subclass deficiency, one patient with PRKDC mutation with IgG subclass deficiency, two patients with severe combined immunodeficiency (SCID; one of them was enrolled before hematopoietic stem cell transplantation (HSCT), and the other was enrolled four years after HSCT), three patients with Nijmegen breakage syndrome (NBS), three patients with ataxia–telangiectasia (A-T). Doses of Ig were individualized and were within 0.2–0.8 g/kg (Table S2). In all cases, substitution therapy had started before the initiation of the current study. Patients with hyper-IgE syndrome, Omenn syndrome, Netherton syndrome, WAS, which are associated with an elevated level of IgE, were not included in the study because they were not represented in our database.

During the study, a detailed medical history of the patients was collected, in particular: (1) a documented diagnosis of allergic disease performed by a trained allergist/pulmonologist, (2) family history of allergic diseases, (3) recurrent respiratory tract infections, (4) skin eczema, (5) medications taken, especially antihistamine drugs, (6) weight and/or height deficiency, (7) place of residence (village, town, city).

The following parameters were assessed by venous blood analysis: (1) concentration of sIgE against 18 food allergens: egg white, egg yolk, cow’s milk, alpha-lactalbumin, beta-lactoglobulin, casein, bovine serum albumin (BSA), codfish, flour mix, rice, soybean, peanut, hazelnut, carrot, potato, apple, cacao, chicken; (2) concentration of sIgE against 11 inhalant allergens: 6 grass mix, birch pollen, mugwort pollen, *Dermatophagoides pteronyssinus*, *Dermatophagoides farinae*, cat epithelia, horse epithelia, dog epithelia, *Cladosporium herbarum*, *Aspergillus fumigatus*, *Alternaria alternata*; (3) IgG concentration; (4) blood eosinophilia; (5) total IgE level.

Clinical symptoms were assessed by a questionnaire including questions on inhalant allergy: sneezing, rhinorrhea, nose/eyes itching, need to rub nose/eyes, watery eyes, nasal obstruction, with or without facial itching, nasal congestion, reduced sense of smell, trouble breathing with mouth closed. Symptoms of IgE-mediated food allergy (like pruritus, flushing, urticaria, angioedema, conjunctival injection, lacrimation, periorbital edema, oropharyngeal symptoms, allergy symptoms from the respiratory tract, gastrointestinal symptoms like nausea/vomiting, abdominal cramping, bloating, diarrhea, signs of anaphylaxis) were assessed separately. Assessment of asthma and atopic dermatitis was mainly based on specialists’ consultations obtained from medical documentation.

sIgE were measured by quantitative enzyme immunoassay—the Polycheck ^®^ test (Biocheck GmbH, Münster, Germany), according to the manufacturer’s instructions. The threshold for a positive result for sIgE, which was indicative of sensitization, was ≥0.35 kUa/L. Subjects with at least one positive sIgE were considered to be sensitized. IgG and total IgE concentrations were measured by immunoturbidimetry. The total IgG and IgE reference ranges depended on the age of the individual. Elevated total IgE was defined as total IgE concentration above the age-related reference ranges.

Statistical analysis of the data was conducted using the spreadsheet of Microsoft Office Excel (Microsoft Corp., Washington, DC, USA) and Statistica v.13—for qualitative data—Pearson’s chi-squared. The significance level was accepted as α = 0,05. A *p*-value less than 0.05 was considered statistically significant.

## 3. Results

### 3.1. Allergen-Specific IgE

sIgE ≥ 0.35 kUa/L were detected in the sera of 50.0% (*n* = 36) of the PID patients (*n* = 72), which correlated (*p* < 0.0001) with the presence of clinical symptoms of inhalant and/or food allergy (Table S3). During the period of the study, 61.11% (*n* = 44) of the children with PID reported subjective symptoms of allergy, and in this group, 77.27% (*n* = 34) tested positive for sIgE. In most cases, there was a correlation between reported symptoms and positive sIgE. In 17 patients, the correlation was more evident, notably if there were symptoms of inhalant allergy (*n* = 12).

sIgE concentrations in the majority of allergic patients (*n* = 20; 27.78% of PID patients) ranged from 0.35 to 3.5 kU/L, while in 22.22% (*n* = 16) of the patients, sIgE concentration was ≥3.5 kU/L (Table 1 and Table S4). sIgE against only one allergen was observed in 22.22% (*n* = 16) of the PID patients, while in 18.06% (*n* = 13), sIgE against three and more allergens were observed. In most cases, sIgE were against food allergens (*n* = 18; 25%), whereas in 9 children (12.5%), they were only against inhalant allergens, and in other 9 children (12.5%) they were against both inhalant and food allergens.

**Figure 1:**
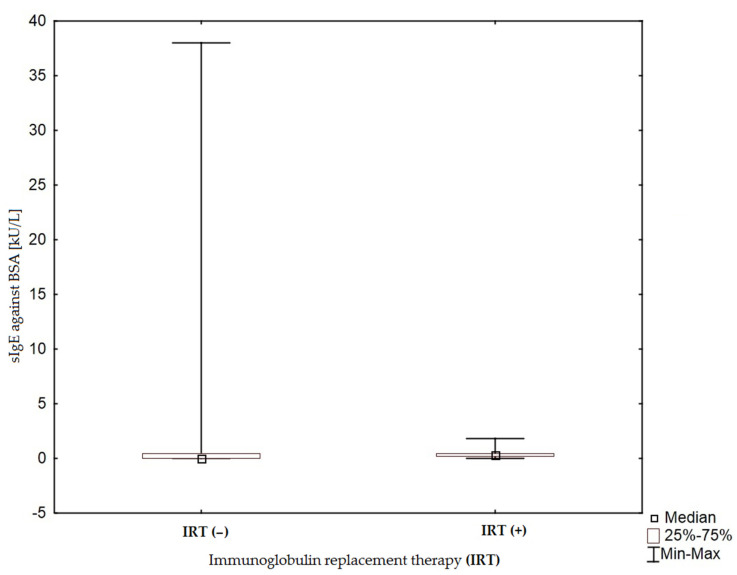
Concentration of sIgE against BSA (bovine serum albumin) in PID patients during immunoglobulin replacement therapy (IRT+) and not receiving IRT (IRT−) (*p* = 0.03).

The presence of sIgE (≥0.35 kU/L) against BSA in 33.33% (*n* = 24) of patients with PID was an interesting finding. In most of these cases (*n* = 20; 83.33%), it correlated with clinical symptoms of milk allergy at the time of the study or in the past. sIgE against BSA showed low values (<0.7 kU/L; *n* = 12). A higher concentration of sIgE against BSA was observed in patients without Ig replacement therapy than in patients receiving Ig substitution therapy, and the difference between these two groups was statistically significant (*p* = 0.03; Figure 1).

Twenty-one (29.17%) patients showed chronic or recurrent skin eczema, and the majority of them (*n* = 16) had a significantly increased sIgE level (*p* = 0.003).

There was no significant correlation between the prevalence of recurrent respiratory tract infections and the serum sIgE level (*p* = 0.55).

The levels of sIgE against inhalant allergens correlated with an elevated total IgE (*p* = 0.0002). However, no correlation with blood eosinophilia (*p* = 0.85), recurrent respiratory tract infections (*p* = 0.23), family history of allergic diseases (*p* = 0.99) was observed. There was no significant difference between males and females (*p* = 0.39).

The levels of sIgE against food allergens did not correlate with eosinophilia (*p* = 0.31), total IgE (*p* = 0.14), weight deficiency (*p* = 0.87), height deficiency (*p* = 0.65), positive family history of allergy (*p* = 0.53). There was no effect of the treatment with antihistamine medications (*p* = 0.54).

Although 72.22% (*n* = 26) of the patients with increased sIgE were males, the difference between the sexes was not significant (*p* = 0.14). In addition, there was no significant difference between different groups of PID (Table 2).

An increased sIgE level was observed in 47.17% (25/53) of patients not receiving IRT and in 57.89% (11/19) of patients undergoing IRT, but the difference between these two groups was not statistically significant (*p* = 0.42).

**Figure 2:**
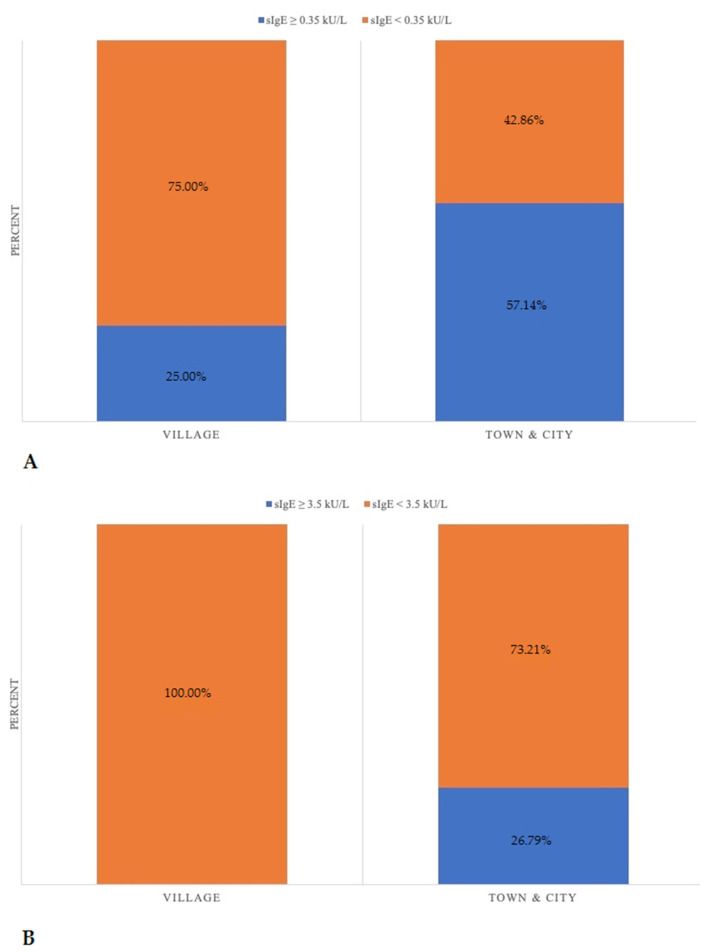
(**A**). sIgE level depending on the living place (*p* = 0.047). (**B**). Prevalence of allergen-specific IgE concentration ≥ 3.5 kU/L depending on the living place (village vs. town and city; *p* = 0.047).

A statistically significant difference (*p* = 0.047) was observed with regard to rural or urban residence (Figure 2): 25% of patients living in the countryside had increased sIgE. Increased sIgE was found in the serum of 57.14% of children living in an urban area, while 26.79% of children from urban environments showed an sIgE level ≥ 3.5 kU/L, and none of the individuals from the countryside had sIgE levels ≥ 3.5 kU/L (*p* = 0.047; Figure 2B).

### 3.2. Total IgE Level

The total IgE level was normal in the majority (69.44%, *n* = 25) of the 36 children with increased (≥0.35 kU/L) sIgE. On the other hand, 84.62% (*n* = 11) of the 13 patients (18.06% of all 72 recruited patients) with an elevated total IgE level had increased sIgE concentration as well. There was a statistically significant correlation between sIgE and total IgE levels (*p* = 0.014). Furthermore, an elevated level of total IgE correlated with clinical symptoms of allergy in this particular group (*p* = 0.004; Table S3) but did not correlate with skin eczema (*p* = 0.31).

**Figure 3:**
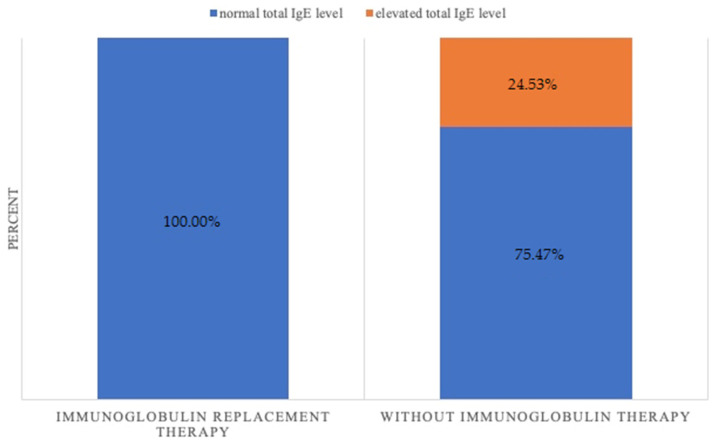
Total IgE level in relation to immunoglobulin replacement therapy (*p* = 0.04).

There was a statistically significant difference (*p* = 0.04) between PID patients treated with Ig and PID patients who were not undergoing Ig therapy (Figure 3). While the total IgE level was elevated in 24.53% (*n* = 13) of patients not receiving IRT, such deviation was not observed in those patients (*n* = 0) who were undergoing IRT.

### 3.3. Blood Eosinophilia

Increased counts of eosinophils were observed in the blood of 15.28% (*n* = 11) of the 72 patients included in the study. Eosinophilia was slightly more often observed in females (*n* = 5; 19.23% of all females) than in males (*n* = 6; 13.04% of all males), but the difference was not statistically significant (*p* = 0.48). The counts of eosinophils did not correlate with sIgE presence (*p* = 0.74), clinical symptoms of allergy (Tables S3 and S5; *p* = 0.62), skin eczema (*p* = 0.85), recurrent respiratory tract infections (*p* = 0.29), or total IgE level (*p* = 0.66).

### 3.4. Clinical Manifestations

Of the 72 patients included in the study, 18.06% were previously diagnosed by a trained allergist/pulmonologist with asthma, 20.84% with AR, 23.61% with FA, and 25% with AD; urticaria occurred in 2.78% of the patients, AC in 1.39%, and drug-induced anaphylaxis in 1.39% (Table S5). Previous diagnoses were obtained from the patients’ medical records.

## 4. Discussion

IgE plays a key role in the pathogenesis of allergic diseases, especially in mast cell/basophil activation, as well as in antigen/allergen presentation. The level of total IgE was considered in early studies as a marker to identify allergic subjects, but it became evident that total IgE levels cannot confirm an allergy status in a patient [14,15].

Performing serological diagnostics in patients with PID presents some challenges. For example, in patients with a common variable immunodeficiency (CVID), who show extremely low levels of IgE, traditional methods of sIgE measurement may provide false-negative results. In such conditions, sensitization should be confirmed using other methods, for example, bronchial provocation tests with allergens, etc. [16]. In a study conducted by Lawrence et al., an undetectable IgE level appeared in 75.6% (95% CI, 65.6–85.7%) of patients with CVID, and allergen sIgE was not detectable in 96.5% of patients with CVID [17]. Three patients with CVID participated in our study, and sIgE (but not total IgE) correlated with clinical symptoms of allergy (Table 2). However, it is worth adding that the sIgE level was between 0.35 and 0.70 kU/L in these patients, and the correlation was not confirmed by an oral food challenge.

According to some studies, the cut-off level of 0.35 kUa/L for allergen-specific IgE might be insufficient to predict clinical reactivity [18,19,20,21,22]. For example, in the study by Garcia-Ara et al., it was shown that different cut-off points for specific IgE levels for milk and casein, which indicated clinical reactivity, were found at different ages. The specific IgE levels that were predictors of clinical reactivity (positive predictive value ≥ 90%), grew as the age of the infants increased: 1.5, 6, and 14 kUA/L for milk for patients in the age ranges of 13–18 and 19–24 months and in the third year, respectively [18]. However, it is worth noticing that this study was not conducted among children with PID. The application of the predictive values of a diagnostic test changes with the disease prevalence, and consequently, these values are not valid for populations with a different prevalence [18]. In our study, an sIgE concentration between 0.35 kU/L and 3.5 kU/L was detected among 27.78% (*n* = 20) of the patients with PID, while 22.22% (*n* = 16) of the study group had an sIgE level above 3.5 kU/L. Nevertheless, we are aware that the utility of, especially, food-specific IgE concentrations in predicting symptomatic food allergies in the context of IEI patients may be questionable, and further studies including, e.g., oral food challenges are needed. Defining the cut-off values for specific IgE among patients with immunodeficiency would be helpful in clinical practice.

Allergy tests, both skin testing and in vitro sIgE tests, must always be interpreted in the context of the patient’s specific clinical history, because a “positive” test does not always entail clinical allergy [23,24,25]. The definitive diagnosis of an IgE-mediated allergy requires the detection of specific IgE and knowledge of the history of allergy on exposure to that allergen [15]. It is not uncommon to find allergen-specific IgE in the serum of people who do not report allergic symptoms [26].

The likelihood that a positive sIgE test result will correlate with clinical symptoms is influenced by the degree of positivity, the type of allergen, and the patient’s clinical history. Venom- and food-specific IgE have been reported in up to 25% and 60% of the general population, respectively [26,27,28]. In food-allergic subjects, the test sensitivity can be improved by using higher cutoffs, which, however, are food-specific. The predictive values also vary with age. Younger children and infants show symptoms while presenting lower sIgE levels compared with older children [18]. Patients with higher sIgE levels are more likely to develop symptoms upon exposure to an allergen than patients with lower sIgE levels. However, strongly positive tests do not necessarily predict the severity of allergic symptoms (anaphylaxis) [29]. Regrettably, the threshold levels have not been determined for most allergens.

An elevated level IgE is often associated with a number of PIDs, such as hyper IgE syndrome, WAS, Netherton syndrome, IPEX syndrome, Omenn syndrome [30]. One phenotype of complete DiGeorge syndrome has oligoclonal T cell expansion with elevated IgE levels. The pathophysiological role of increased IgE in these disorders is unclear. However, patients with these disorders were not included in this study. Children with hypogammaglobulinemia (*n* = 20) involving one or more main classes of immunoglobulins as well as patients with IgG subclass deficiency (*n* = 20) constituted the majority of the study group. The prevalence of sIgE and clinical symptoms of allergy varied between the types of PID (Table 2), affecting 50% (sIgE presence) and 61.11% (self-reported allergy symptoms) of the patients in the study group, respectively.

In the USIDNET study, the prevalence of FA as well as AD in PID patients from the USA was found to be lower than in the general population (FA, 2.5%; AD, 10.7%). However, the authors indicated that FA was more commonly reported in patients with specific PIDs, compared to what would be expected in the general population, i.e., in the presence of combined immunodeficiencies (33.3%), sIgAD (25%), CD40 ligand deficiency (7.7%), primary hypogammaglobulinemia (7.1%), hyper IgE syndrome (6.3%) [11]. AD was most commonly reported in patients with a deficiency of the nuclear factor κB essential modulator (62.5%), WAS (41.5%), combined immunodeficiency (33.3%), selective IgM deficiency (33.3%), and hyper IgE syndrome (25%) [11].

Although the problem of the coexistence of allergy and PID diseases has been a subject of investigation in a number of studies, the results are largely inconsistent. While in an Iranian study involving patients with sIgAD, atopic eczema was observed in 52% of the patients [12], in Brazil, it affected only 2.38% of the patients [31], and in a study conducted in Sweden, a statistically significant correlation between AD and sIgAD was not observed [13]. In a study conducted by Dadkhah et al., 20% of Iranian patients with hypogammaglobulinemia additionally suffered from asthma, 22% of patients were diagnosed with AR, and 9% with AD [32]. A study from Kuwait confirmed that 19% of PID patients suffered from AD [33]. A 2020 study conducted in a Polish city in patients with Ab deficiencies revealed that the total level of IgE was elevated in 21% of the patients, and sIgE against inhalant allergens was found in 21% of the patients. The most common clinical manifestations included asthma (66.7% patients), AD (22.8%), and AR (12.3%) [34]. In our study, a comparable, elevated total IgE level (23.53%; Table 2) was observed in patients with PAD. The presence of sIgE was much more common (47.06%) in our patients with PAD, but it is worth mentioning that in our study, we used a larger test kit with more allergens, and different laboratory methods were applied in both studies (ImmunoCAP vs. Polycheck).

In the group of patients with PID, differences with regard to the place of residence and an increased prevalence of allergy in patients residing in urban areas were observed, which mirrors the results of epidemiological studies involving the general population [35]. This is definitely an interesting observation, and more studies concerning the impact of environmental factors on the prevalence of serum sIgE and the progress of allergy in this particular group of patients are needed.

The fact that the values of the total IgE level were normal in PID patients undergoing IRT is an interesting observation that is confirmed by some other studies [36,37]. Durandy et al. treated a cohort of children with recurrent infections with low doses of Ig and observed that in several allergic patients from this group, in whom serum IgE concentrations were high, the serum levels of IgE were normalized after 3 to 4 months of Ig therapy [38]. A study conducted by Jee et al. in patients with AD and an elevated total IgE concentration demonstrated that intravenous immune globulin (IVIg) therapy (2 g/kg/month) had an impact on IgE levels: the levels declined during IVIg treatment but returned to their initial values 6 months after treatment [37]. Noh et al. investigated 41 children given a single dose of IVIg and observed that in 40% of the subjects there was a significant improvement associated with a decrease in serum IgE [39]. This effect may be associated with the immunomodulatory effect of IVIg or stem from a downregulation of the factors driving IgE production [39,40]. The impact of substitutional doses of immunoglobulins on total IgE and specific IgE in PID patients in a long-time perspective requires further research and should involve a larger study group. However, it is worth noticing that few patients in the Ig replacement therapy group (e.g., with X-linked agammaglobulinemia, CVID) might have a deficiency of other antibody classes, including IgE; however, there were also patients with other conditions, such as IgG subclass deficiency/Nijmegen breakage syndrome/ataxia–telangiectasia, in that group (IRT+).

Another point to be addressed regards patients with reported symptoms of allergy without positive (≥0.35 kU/L) sIgE (*n* = 10). Two patients from that group were receiving Ig replacement therapy at a stable dose during the entire study, two had an sIgE concentration between 0.15 kU/L and 0.35 kU/L against cat dander, and another two against birch pollen with airway allergic symptoms. The possibility of non-IgE-mediated mechanisms remains to be investigated.

The main limitations of this study was the use of a single method for confirming patients’ sensitization, without provocation tests and assessment of asthma phenotypes. These issues will be addressed in follow-up studies.

In IEI, an abnormally functioning immune system often does not protect the organism from infections and permits abnormal and excessive hypersensitivity reactions. The development of allergies in IEI is caused by the disruption of the complex balance between effector and regulatory cells in the immune system [6]. The potential mechanisms leading to such dysregulation include failure of central thymic tolerance, imbalance between effector and regulatory T-cell functions, failure in the production of counter-regulating interferon-gamma (IFN-γ) [6,41]. Possible differences in microbial colonization and infection patterns are additional factors of interest.

## 5. Conclusions

This study revealed that the prevalence of allergic diseases and atopy in the pediatric population with IEI is high. PID patients should be carefully monitored with regard to their risk of allergy. The study showed that serum sIgE and total IgE together might be a plausible diagnostic tool for these patients. However, for patients with IRT, the assessment of total IgE is not useful in the context of allergy. Further, more detailed studies including patients with increased levels of sIgE are needed.

## Figures and Tables

**Table 1:** Characteristics of patients with sIgE concentration ≥ 3.5 kU/L.

Patient No	Age	Gender	PID Type	Previous Diagnosis of Allergic Disease	Reported Allergy Symptoms	sIgE [kU/L]	Total IgE [IU/mL]	Correlation between sIgE and Allergy Symptoms	Eosinophil Count [10^3^/uL]	Antihistamines
1	8	M	IgM, IgG subclass deficiency	A, AR, FA	Nasal obstruction Sneezing Nose/eyes itching Trouble breathing with mouth closed Recurrent bronchitis	6 Grass mix = 2.9 *D. pteronyssinus* > 100 *D. farinae* > 100 Cat = 33.0 Dog = 0.89 *A. alternata* = 12	587 [0.5–393]	Yes	1.77	Yes
2	14	M	IgM deficiency	AR, FA	Rhinorrhea Diarrhea	*D. pteronyssinus* = 13.0 *D. farinae* = 65.0 Cat = 0.81 BSA = 0.5	207 [1.9–170]	Possible	0.12	No
3	13	M	IgA deficiency	AD	Nose/eyes itching Eczema Pruritus	Horse = 0.53 Cow’s milk = 2.0 BSA = 38.0	34,2 [1.9–170]	Possible	0.17	No
4	7	M	IgM and IgG subclass deficiency	No	Sneezing	*D. pteronyssinus* > 100 *D. farinae* > 100 Cat = 1.2 Dog = 2.2 Horse = 0.45	328 [0.5–393]	Yes	3.75	No
5	6	F	IgG subclass deficiency	A, AD, FA	Eczema Pruritus Sneezing Nose/eyes itching Trouble breathing with mouth closed	6 Grass mix > 100 Birch pollen > 100 Mugwort pollen = 0.86 *D. pteronyssinus* = 4.5 *D. farinae* = 48 Cat = 0.27 Dog = 74 *C. herbarum* = 1.9 *A. alternata* = 5.0 Egg white = 0.94 Cow’s milk = 0.94 Casein = 0.36 Flour Mix 0.46 Rice = 0.86 Peanut = 54.0 Hazelnut = 0.91 Carrot = 0.77 Potato = 1.8 Apple = 0.99	823.5 [0.5–393]	Yes	0.32	Yes
6	12	F	IgG subclass deficiency	AR	Sneezing Nose/eyes itching Watery eyes Need to rub eyes/nose Nasal obstruction	6 Grass mix > 100 Birch pollen = 3.5 Mugwort pollen = 0.81 *D. pteronyssinus* = 1.3 *D. farinae* = 11 Cat = 4.1 Dog = 0.6 *A. alternata* = 1.2 Flour Mix = 0.43 Rice = 0.48 Carrot = 0.50	466 [1.9–170]	Yes	0.17	Yes
7	6	M	IgA deficiency	A, AR	Sneezing Nose/eyes itching Watery eyes Need to rub eyes/nose Nasal obstruction Nasal congestion Rhinorrhea Abdominal cramping Recurrent bronchitis	*D. pteronyssinus* > 100 *D. farinae* > 100 BSA = 4.8 Cow’s milk = 0.44	699 [0.5–393]	Yes	0.35	Yes
8	4	F	IgG subclass deficiency	A	Sneezing Nose/eyes itching Watery eyes Need to rub eyes/nose Recurrent bronchitis	Birch pollen = 1.3 Cat = 15	38.4 [0.4–351]	Yes	0.72	Yes
9	6	F	IgA and IgG subclass deficiency	AD, AR, FA	Sneezing Nose/eyes itching Watery eyes Need to rub eyes/nose Rhinorrhea Nasal obstruction Eczema	6 Grass mix = 60 Birch pollen = 1.7	134 [<90]	Yes	0.12	Yes
10	10	F	IgG subclass deficiency	AR	Rhinorrhea	6 Grass mix > 100 Birch pollen = 0.63 Mugwort pollen = 0.64	227 [<200]	Yes	0.22	No
11	16	M	IgM deficiency	AD, AR	Eczema Rhinorrhea	6 Grass mix > 100 Birch pollen = 0.50 Mugwort pollen = 0.51 Cow’s milk = 0.54 BSA = 4.9	1157 [1.5–100]	Possible	0.17	Yes
12	9	M	PRKDC mutation, IgG subclass deficiency	AD, AR, FA	Eczema Nasal congestion Cough Sneezing Nose/eyes itching	6 Grass mix = 4.1 Birch pollen = 0.36 Mugwort pollen = 0.88 Cat = 0.96 Dog = 0.63	131 [0.5–393]	Yes	0.23	Yes
13	3	F	Complement deficiency	AD	Eczema Pruritus Excoriatio Rhinorrhea Cough	Dog = 0.37 Horse = 0.36 BSA = 9.9 Cow’s milk = 0.73	10.5 [<60]	Possible	0.2	No
14	2	M	Phagocyte number/function deficiency	U	Urticaria	Cow’s milk = 0.77 BSA = 7.0	17.3 [<60]	Possible	0.22	No
15	7	F	Congenital asplenia	AR	Rhinorrhea Nose/eyes itching	6 Grass mix = 4.0 Horse = 0.72 *A. alternata* = 9.4	47.3 [0.5–393]	Yes	0.27	Yes
16	10	F	Lymphocyte T deficiency	U	Urticaria Flushing	6 Grass mix = 2.7 Birch pollen = 0.36 Mugwort pollen = 0.58 *D. pteronyssinus* = 0.81 *D. farinae* = 0.94 Cow’s milk = 0.52 BSA = 4.4 Flour Mix = 0.37 Rice = 0.44 Carrot = 0.39 Potato = 0.93 Apple = 1.00	908 [1.9–170]	Possible	0.19	Yes

Abbreviations: A—asthma; AC—allergic conjunctivitis; AD—atopic dermatitis; AR—allergic rhinitis; BSA—bovine serum albumin; F—female; FA—food allergy; Ig—immunoglobulin; M—male; No—number; PID—primary immunodeficiencies; PRKDC—Protein Kinase, DNA-Activated, Catalytic Subunit; U—urticaria.

**Table 2:** Prevalence of sensitization to food allergens, skin eczema, clinical symptoms of allergy, elevated total IgE and sIgE levels in the recruited patients with PID.

Type of PID (*n* = 72)	Clinical Symptoms of Allergy (*n* = 44)	sIgE ≥ 0.35 kUa/L (*n* = 36)	Elevated Total IgE (*n* = 13)	Sensitization to Food Allergens (sIgE ≥ 0.35 kUa/L) + Clinical Symptoms (*n* = 23)	Skin Eczema (*n* = 21)
Selected Parameter					
SCID (*n* = 2)	*n* = 0 (0.00%)	*n* = 1 (50.00%)	*n* = 0 (0.00%)	*n* = 0 (0.00%)	*n* = 0 (0.00%)
Ataxia-telangiectasia (*n* = 4)	*n* = 3 (75.00%)	*n* = 3 (75.00%)	*n* = 0 (0.00%)	*n* = 2 (50.00%)	*n* = 1 (25.00%)
Nijmegen syndrome (*n* = 3)	*n* = 1 (33.33%)	*n* = 1 (33.33%)	*n* = 0 (0.00%)	*n* = 1 (33.33%)	*n* = 1 (33.33%)
DiGeorge syndrome (*n* = 2)	*n* = 1 (50.00%)	*n* = 1 (50.00%)	*n* = 0 (0.00%)	*n* = 1 (50.00%)	*n* = 0 (0.00%)
Kabuki syndrome (*n* = 1)	*n* = 0 (0.00%)	*n* = 0 (0.00%)	*n* = 0 (0.00%)	*n* = 0 (0.00%)	*n* = 0 (0.00%)
PRKDC mutation (*n* = 1)	*n* = 1 (100.00%)	*n* = 1 (100.00%)	*n* = 0 (0.00%)	*n* = 0 (0.00%)	*n* = 1 (100.00%)
Predominantly Ab deficiency (*n* = 51):	*n* = 31 (60.78%)	*n* = 24 (47.06%)	*n* = 12 (23.53%)	*n* = 15 (29.41%)	*n* = 16 (31.37%)
CVID (*n* = 3)	*n* = 2 (66.67%)	*n* = 2 (66.67%)	*n* = 0 (0.00%)	*n* = 2 (66.67%)	*n* = 1 (33.33%)
X-linked agammaglobulinemia (*n* = 1)	*n* = 1 (100.00%)	*n* = 1 (100.00%)	*n* = 0 (0.00%)	*n* = 1 (100.00%)	*n* = 1 (100.00%)
other hypo-gammaglobulinemia’s (*n* = 20)	*n* = 12 (60.00%)	*n* = 10 (50.00%)	*n* = 4 (20.00%)	*n* = 4 (20.00%)	*n* = 6 (30.00%)
IgG subclass deficiency (*n* = 20)	*n* = 12 (60.00%)	*n* = 7 (35.00%)	*n* = 6 (30.00%)	*n* = 5 (25.00%)	*n* = 7 (35.00%)
selective IgA deficiency (*n* = 7)	*n* = 4 (57.14%)	*n* = 4 (57.14%)	*n* = 2 (28.57%)	*n* = 3 (42.86%)	*n* = 1 (14.29%)
Congenital defects of phagocyte number, function or both (*n* = 3)	*n* = 2 (66.67%)	*n* = 1 (33.33%)	*n* = 0 (0.00%)	*n* = 1 (33.33%)	*n* = 0 (0.00%)
Complement deficiency (*n* = 2)	*n* = 2 (100.00%)	*n* = 2 (100.00%)	*n* = 0 (0.00%)	*n* = 2 (100.00%)	*n* = 1 (50.00%)
Others (*n* = 3)	*n* = 3 (100.00%)	*n* = 2 (66.67%)	*n* = 1 (33.33%)	*n* = 1 (33.33%)	*n* = 1 (33.33%)

Abbreviations: Ab—antibody; CVID—common variable immunodeficiency; *n*—number of cases; sIgE—allergen-specific immunoglobulin class E; PID—primary immunodeficiencies; PRKDC—Protein Kinase, DNA-Activated, Catalytic Subunit; SCID—severe combined immunodeficiency; X-linked—gene is located in the X chromosome.

## Data Availability

The authors confirm that the data supporting the findings of this study are available within the article and its Supplementary Materials.

## Supplementary Materials

The following are available online at www.mdpi.com/article/10.3390/children9040466/s1, Table S1: Patients’ baseline characteristics according to PID classification. Table S2: Characteristics of patients during immunoglobulin replacement therapy (*n* = 19). Table S3: Characteristics of patients with positive (≥0.35 kU/L) sIgE against allergens. Table S4: Number of patients with the maximum sIgE concentration against allergens. Table S5: Manifestation of allergy in patients with PID, expressed as previously diagnosed asthma, food allergy, allergic conjunctivitis, atopic dermatitis, allergic rhinitis, or urticaria.

## Abbreviations

A	asthma
Ab	antibody(ies)
AC	allergic conjunctivitis
AD	atopic dermatitis
AR	allergic rhinitis
A-T	ataxia–telangiectasia
BSA	bovine serum albumin
CVID	common variable immunodeficiency
FA	food allergy
HSCT	hematopoietic stem cell transplantation
ICD-10	International Statistical Classification of Diseases and Related Health Problems
IEI	inborn errors of immunity
IFN-γ	interferon-gamma
IgE/G/A/M	immunoglobulin(s) class E/G/A/M
IPEX syndrome	immunodysregulation, polyendocrinopathy, enteropathy X-linked syndrome
IRT	immunoglobulin replacement therapy
IUIS	International Union of Immunodeficiency Societies
*n*	number
NBS	Nijmegen breakage syndrome
NHANES	The National Health and Nutrition Examination Survey
PAD	predominantly antibody deficiencies
PID	primary immunodeficiency
PRKDC	Protein Kinase, DNA-Activated, Catalytic Subunit
sIgE	allergen-specific immunoglobulin class E
sIgAD	selective IgA deficiency
SCID	severe combined immunodeficiency
SPT	skin prick test
U	urticaria
USIDNET	US Immunodeficiency Network
WAS	Wiskott–Aldrich syndrome
X-linked	gene is located in the X chromosome
