# Supplementary material for: Serum Allergen-Specific IgE among Pediatric Patients with Primary Immunodeficiency
Source: Children (Basel). 2022 Mar 25;9(4):466. doi: 10.3390/children9040466 (PMC9029572; doi:10.3390/children9040466)
Supplement: Supplementary file 1 [file children-09-00466-s001.zip › Children new Table s3 10.pdf]

Table S3. Characteristics of patients with positive (>=0.35 kU/L) sIgE against allergens.

Patient No	Age	Gender	PID type	Previous diagnosis of allergic disease	Reported allergy symptoms	sIgE [kU/L]	Total IgE [IU/ml]	Correlation between sIgE and allergy symptoms	Eosinophil count [10^3/uL]	Antihistamines
1	15	M	X-linked agammaglobulinemia	No	Eczema Pruritus	BSA = 0.43	15.5 [1.5-100]	Not excluded	0.07	No
2	8	M	IgM, IgG subclass deficiency	A, AR, FA	Nasal obstruction Sneezing Nose/eyes itching Trouble breathing with mouth closed Recurrent bronchitis Allergic asthma	6 Grass mix = 2.9 D.pteronyssinus >100 D. farinae >100 Cat = 33.0 Dog = 0.89 A.alternata = 12	587 [0.5-393]	Yes	1,77	Yes
3	2	M	IgG subclass deficiency	No	Eczema Pruritus	D. farinae = 0.37 BSA = 0.45	14 [0.4-351]	Possible	0.35	No
4	14	M	IgM deficiency	AR, FA	Rhinorrhea Diarrhoea	D.pteronyssinus = 13.0 D. farinae =65.0 Cat = 0.81 BSA = 0.5	207 [1.9-170]	Yes	0,12	No
5	14	M	CVID	No	Eczema	BSA = 0.42	8.1 [1,9-170]	Not excluded	0.1	No
6	12	M	CVID	No	Diarrhoea	BSA = 0.4	9.2 [1.9-170]	Not excluded	0.08	No
7	13	M	IgA deficiency	AD	Nose/eyes itching Eczema Pruritus	Horse = 0.53 Cow's milk = 2.0 BSA = 38.0	34,2 [1.9-170]	Possible	0,17	No
8	7	M	IgM and IgG subclass deficiency	No	Sneezing	D.pteronyssinus >100 D. farinae >100 Cat = 1.2 Dog = 2.2 Horse = 0.45	328 [0.5-393]	Yes	3.75	No
9	6	F	IgG subclass deficiency	A, AD, FA	Eczema Pruritus Allergic asthma Sneezing Nose/eyes itching	6 Grass mix >100 Birch pollen >100 Mugwort pollen = 0.86 D.pteronyssinus =4.5	823.5 [0.5-393]	Yes	0,32	Yes

					Trouble breathing with mouth closed	D. farinae =48<br>Cat = 0.27<br>Dog = 74<br>C.herbarum = 1.9<br>A.alternata = 5.0<br>Egg white = 0.94<br>Cow's milk = 0.94<br>Casein = 0.36<br>Flour Mix 0.46<br>Rice = 0.86<br>Peanut = 54.0<br>Hazelnut = 0.91<br>Carrot = 0.77<br>Potato = 1.8<br>Apple = 0.99				
10	9	M	IgG deficiency	A	Recurrent bronchitis<br>Cough<br>Nasal congesition<br>Trouble breathing<br>Milk allergy in the past, nondairy diet in the past	BSA = 0.51	203.2 [0.5-393]	Not excluded	0,17	No
11	15	M	IgA and IgM deficiency	No	Abdominal cramping, bloating	BSA = 0.72	16.8 [1.5-100]	Not excluded	0.05	No
12	12	F	IgG subclass deficiency	AR	Sneezing<br>Nose/eyes itching<br>Watery eyes<br>Need to rub eyes/nose<br>Nasal obstruction	6 Grass mix >100<br>Birch pollen =3.5<br>Mugwort pollen = 0.81<br>D.pteronyssinus = 1.3<br>D. farinae = 11<br>Cat = 4.1<br>Dog = 0.6<br>A.alternata = 1.2<br>Flour Mix = 0.43<br>Rice = 0.48<br>Carrot = 0.50	466 [1.9-170]	Yes	0,17	Yes
13	6	M	IgA deficiency	A, AR	Sneezing<br>Nose/eyes itching<br>Watery eyes<br>Need to rub eyes/nose	D.pteronyssinus >100<br>D. farinae >100<br>BSA = 4.8<br>Cow's milk = 0.44	699 [0.5-393]	Yes	0,35	Yes

					Nasal obstruction<br>Nasal congestion<br>Rhinorrhoea<br>Abdominal cramping<br>Recurrent bronchitis<br>Allergic asthma					
14	5	M	IgG subclass deficiency	AD	Eczema<br>Rhinorrhoea<br>Trouble breathing<br>Cough	BSA = 0.42	594 [0.5-393]	Not excluded	0,12	No
15	4	F	IgG subclass deficiency	A	Sneezing<br>Nose/eyes itching<br>Watery eyes<br>Need to rub eyes/nose<br>Allergic asthma<br>Recurrent bronchitis	Birch pollen =1.3<br>Cat = 15	38.4 [0.4-351]	Yes	0,72	Yes
16	8	M	IgM and IgG subclass deficiency	A, AR, FA	Rhinorrhoea<br>Cough<br>Nasal congestion<br>Trouble breathing with mouth closed<br>Recurrent bronchitis<br>Eczema	D. farinae = 1.1	19.5 [0.5-393]	Yes	0,21	No
17	2,5	M	IgG subclass deficiency	AD, FA	Eczema<br>Milk allergy in the past<br>Nondairy diet in the past	BSA = 0.9	35.6 [0.4-351]	Possible	0,91	No
18	4	M	IgA deficiency	No	Cough<br>Nasal congestion<br>Rhinorrhoea	BSA = 2.2	<10 [<60]	Unlikely	0.26	No
19	6	F	IgA and IgG subclass deficiency	AD, AR, FA	Sneezing<br>Nose/eyes itching<br>Watery eyes<br>Need to rub eyes/nose<br>Rhinorrhoea	6 Grass mix = 60<br>Birch pollen =1.7	134 [<90]	Yes	0,12	Yes

					Nasal obstruction Eczema					
20	10	F	IgG subclass deficiency	AR	Rhinitis	6 Grass mix >100 Birch pollen = 0.63 Mugwort pollen = 0.64	227 [<200]	Yes	0,22	No
21	6	M	IgA deficiency	A	Cough Nasal congestion Trouble breathing with mouth closed Recurrent bronchitis Diarrhoea	BSA = 0.89 Cow's milk = 0.41	2347 [<90]	Possible	0,67	No
22	2	M	IgG deficiency	AD, FA	Eczema Nose itching Need to rub nose Nondairy diet	6 Grass mix = 0.35 Birch pollen = 0.93	10 [<60]	Possible	0,18	Yes
23	16	M	IgM deficiency	AD, AR	Eczema Rhinitis	6 Grass mix >100 Birch pollen =0.50 Mugwort pollen = 0.51 Cow's milk = 0.54 BSA = 4.9	1157 [1.5-100]	Yes	0,17	Yes
24	4	M	IgA and IgM deficiency	No	Abdominal cramping	BSA = 2.0	11.6 [0.5-393]	Not excluded	0.12	No
25	5	M	SCID	No	No symptoms	BSA = 0.37	7.5 [0.5-393]	No	0.19	No
26	10	M	Ataxia-telangiectasia	FA	Vomiting Abdominal cramping	BSA = 0.41	<1.5 [0.5-393]	Possible	0,38	No
27	11	F	Ataxia-telangiectasia	No	Eczema	BSA = 0.67	9 [1.9-170]	Not excluded	0.17	No
28	11	M	Ataxia-telangiectasia	No	Rhinorrhoea Cough Trouble breathing with mouth closed Nasal obstruction Sneezing Nose/eyes itching Watery eyes Need to rub eyes/nose	BSA = 0.45	8.6 [1.9-170]	Not excluded	0.08	No

29	9	F	DiGeorge syndrome	FA	Rash Abdominal cramping, bloating Diarrhoea	BSA = 0.46	64,9 [0.5-393]	Possible	0,21	No
30	9	M	PRKDC mutation, IgG subclass deficiency	AD, AR, FA	Eczema Nasal congestion Cough Sneezing Nose/eyes itching	6 Grass mix =4.1 Birch pollen =0.36 Mugwort pollen = 0.88 Cat = 0.96 Dog = 0.63	131 [0,5-393]	Yes	0,23	Yes
31	4	M	Nijmegen breakage syndrome	AD	Eczema Milk allergy in the past	BSA = 1.8 Cow's milk = 0.4	10 [0.5-393]	Possible	0,09	No
32	7	M	Complement deficiency	No	Cough	Apple = 0.68	32.2 [0.5-393]	Possible	0.15	No
33	3	F	Complement deficiency	AD	Eczema Pruritus Excoriatio Rhinorrhoea Cough	Dog = 0.37 Horse = 0.36 BSA = 9.9 Cow's milk = 0.73	10.5 [<60]	Possible	0,2	No
34	2	M	Phagocyte number/ function deficiency	U	Urticaria	Cow's milk = 0.77 BSA = 7.0	17.3 [<60]	Possible	0,22	No
35	7	F	Congenital asplenia	AR	Rhinorrhea Nose/eyes itching	6 Grass mix = 4.0 Horse = 0.72 A.alternata = 9.4	47.3 [0.5-393]	Yes	0,27	Yes
36	10	F	Impaired cellular immunity	U	Urticaria Flushing	6 Grass mix = 2.7 Birch pollen =0.36 Mugwort pollen = 0.58 D.pteronyssinus = 0.81 D. farinae = 0.94 Cow's milk = 0.52 BSA = 4.4 Flour Mix = 0.37 Rice = 0.44 Carrot = 0.39 Potato = 0.93 Apple = 1.00	908 [1.9-170]	Possible	0,19	Yes

Abbreviations: A – asthma; AC – allergic conjunctivitis; AD – atopic dermatitis; AR – allergic rhinitis; BSA – bovine serum albumin; F – female; FA – food allergy; Ig – immunoglobulin; M – male; No – number; PID – primary immunodeficiencies; PRKDC - Protein Kinase, DNA-Activated, Catalytic Subunit; sIgE – specific IgE; U – urticaria.
