# Supplementary material for: Serum Allergen-Specific IgE among Pediatric Patients with Primary Immunodeficiency
Source: Children (Basel). 2022 Mar 25;9(4):466. doi: 10.3390/children9040466 (PMC9029572; doi:10.3390/children9040466)
Supplement: Supplementary file 1 [file children-09-00466-s001.zip › Children new Table S4 21.pdf]

Table S4. Number of patients with the maximum sIgE concentration against allergens (if the patient had multiple sIgE detected, he/she has been classified to the higher class).

Class	sIgE concentration [kU/L]	Number of patients (% of all patients in the study group)
0	<0.35	36 (50.00%)
1	0.35-<0.7	13 (18.06%)
2	0.7-<3.5	7 (9.72%)
3	3.5-<17.5	6 (8.33%)
4	17.5-<50	1 (1.39%)
5	50-<100	2 (2.78%)
6	>=100	7 (9.72%)
