# Supplementary material for: Serum Allergen-Specific IgE among Pediatric Patients with Primary Immunodeficiency
Source: Children (Basel). 2022 Mar 25;9(4):466. doi: 10.3390/children9040466 (PMC9029572; doi:10.3390/children9040466)
Supplement: Supplementary file 1 [file children-09-00466-s001.zip › Children new Table S5 21.pdf]

Table S5. Manifestation of allergy in patients with PID, expressed as previously diagnosed asthma, food allergy, allergic conjunctivitis; atopic dermatitis, allergic rhinitis or urticaria.

Patient No	Age	Gender	PID type	Previous diagnosis of allergic disease	Duration of allergy symptoms	sIgE level: *not detected, **detected, ***positive [kU/L]	IgG level [mg/dl]	Eosinophil count [10^3/uL]	Antihistamines	Ig replacement therapy
1	17	M	IgA deficiency	A, AD, AR	From early childhood	Detected Cat = 0.27 Dog = 0.32	1386	0,09	Yes	No
2	9	F	DiGeorge syndrome	FA	From early childhood	Positive BSA = 0.46	1275	0,21	No	No
3	8	M	IgM, IgG subclass deficiency	A, AR, FA	From early childhood	Positive 6 Grass mix = 2.9 D.pteronyssinus >100 D. farinae >100 Cat = 33.0 Dog = 0.89 A.alternata = 12	614	1,77	Yes	No
4	6	M	IgG subclass deficiency	A, AR, FA	From infancy	Not detected <0.15	637	0,22	Yes	No
5	14	M	IgM deficiency	AR, FA	Few years	Positive D.pteronyssinus = 13.0 D. farinae =65.0 Cat = 0.81 BSA = 0.5	1025	0,12	No	No
6	13	M	IgA deficiency	AD	From infancy	Positive Horse = 0.53 Cow's milk = 2.0 BSA = 38.0	1472,7	0,17	No	No
7	3	M	Transient hypogammaglobulinemia of infancy	FA in the past	Infancy	Not detected <0.15	508	0,17	No	No
8	6	F	IgG subclass deficiency	A, AD, FA	From infancy	Positive 6 Grass mix >100 Birch pollen >100 Mugwort pollen = 0.86 D.pteronyssinus =4.5 D. farinae =48 Cat = 0.27 Dog = 74 C.herbarum = 1.9 A.alternata = 5.0 Egg white = 0.94 Cow's milk = 0.94	964	0,32	Yes	No

						Casein = 0.36<br>Flour Mix 0.46<br>Rice = 0.86<br>Peanut = 54.0<br>Hazelnut = 0.91<br>Carrot = 0.77<br>Potato = 1.8<br>Apple = 0.99				
9	9	M	PRKDC mutation, IgG subclass deficiency	AD, AR, FA	Few years	Positive<br>6 Grass mix =4.1<br>Birch pollen =0.36<br>Mugwort pollen = 0.88<br>Cat = 0.96<br>Dog = 0.63	969	0,23	Yes	Yes
10	1	M	IgG deficiency	AD	From infancy	Not detected <0.15	254	0,11	No	No
11	12	F	IgG subclass deficiency	AR	1 year	Positive<br>6 Grass mix >100<br>Birch pollen =3.5<br>Mugwort pollen = 0.81<br>D.pteronyssinus = 1.3<br>D. farinae = 11<br>Cat = 4.1<br>Dog = 0.6<br>A.alternata = 1.2<br>Flour Mix = 0.43<br>Rice = 0.48<br>Carrot = 0.50	1108	0,17	Yes	No
12	6	M	IgA deficiency	A, AR	2 years	Positive<br>D.pteronyssinus >100<br>D. farinae >100<br>BSA = 4.8<br>Cow's milk = 0.44	1376	0,35	Yes	No
13	5	M	IgG subclass deficiency	AD	From infancy	Positive<br>BSA = 0.42	992	0,12	No	No
14	2	F	IgG subclass deficiency	AD	From infancy	Detected<br>Cat = 0.23<br>Dog = 0.31<br>Egg Yolk = 0,17<br>Hazelnut = 0.2	576	0,76	No	No
15	4	F	IgG subclass deficiency	A	3 years	Positive<br>Birch pollen =1.3<br>Cat = 15	480	0,72	Yes	No
16	4	M	IgG subclass deficiency	AD	From infancy	Not detected <0.15	594	0,12	No	No
17	8	M	IgM and IgG subclass deficiency	A, AR, FA	6 years	Positive<br>D. farinae = 1.1	823	0,21	No	No

18	2,5	M	IgG subclass deficiency	AD, FA	From infancy	Positive BSA = 0.9	509	0,91	No	No
19	7	F	IgG subclass deficiency	AR	2 years	Detected Cat = 0.21	838	0,39	No	No
20	9	M	IgG subclass deficiency	A	From early childhood	Detected Birch pollen =0.17 A.fumigatus = 0.21	619	0,57	No	No
21	13	F	IgG subclass deficiency	AR	Few years	Not Detected <0.15	613	0,04	No	No
22	6	F	IgA and IgG subclass deficiency	AD, AR, FA	From infancy	Positive 6 Grass mix = 60 Birch pollen =1.7	895	0,12	Yes	No
23	10	F	IgG subclass deficiency	AR	Few years	Positive 6 Grass mix >100 Birch pollen = 0.63 Mugwort pollen = 0.64	778	0,22	No	No
24	6	M	IgA deficiency	A	From infancy	Positive BSA = 0.89 Cow's milk = 0.41	1296	0,67	No	No
25	3	F	Complement deficiency	AD	From infancy	Positive Dog = 0.37 Horse = 0.36 BSA = 9.9 Cow's milk = 0.73	939	0,2	No	No
26	9	M	IgG subclass deficiency	A, AD, FA	From infancy	Detected BSA = 0.27	826	0,07	No	No
27	2	M	IgG deficiency	AD, FA	From infancy	Positive 6 Grass mix = 0.35 Birch pollen = 0.93	454	0,18	Yes	No
28	10	F	Impaired cellular immunity	U	Few months	Positive 6 Grass mix = 2.7 Birch pollen =0.36 Mugwort pollen = 0.58 D.pteronyssinus = 0.81 D. farinae = 0.94 Cow's milk = 0.52 BSA = 4.4 Flour Mix = 0.37 Rice = 0.44 Carrot = 0.39 Potato = 0.93 Apple = 1.00	939	0,19	Yes	No
29	16	M	IgM deficiency	AD, AR	From early childhood	Positive 6 Grass mix >100 Birch pollen =0.50	987	0,17	Yes	No

						Mugwort pollen = 0.51<br>Cow's milk = 0.54<br>BSA = 4.9				
30	2	M	Phagocyte number/ function deficiency	U	From infancy	Positive<br>Cow's milk = 0.77<br>BSA = 7.0	683	0,22	No	No
31	4	M	IgG deficiency	AC, AD, FA	From infancy	Not Detected<br><0.15	849	0,73	Yes	Yes
32	10	M	Ataxia-telangiectasia	FA, DIA	From infancy	Positive<br>BSA = 0.41	1018	0,38	No	Yes
33	4	F	Phagocyte number/ function deficiency	FA in the past	Infancy	Detected<br>Cat = 0.27	653	0,08	No	No
34	7	F	IgG subclass deficiency	FA in the past	Infancy	Detected<br>Birch pollen =0.18	751	0,12	No	No
35	7	F	Congenital asplenia	AR	Few years	Positive<br>6 Grass mix = 4.0<br>Horse = 0.72<br>A.alternata = 9.4	1349	0,27	Yes	No
36	9	M	IgG deficiency	A	From early childhood	Positive<br>BSA = 0.51	1145	0,17	No	Yes
37	8	F	IgG subclass deficiency	AD, AR	From early childhood	Detected<br>Birch pollen = 0.18<br>BSA = 0.29	742	0,1	Yes	Yes
38	4	M	Nijmegen breakage syndrome	AD	From infancy	Positive<br>BSA = 1.8<br>Cow's milk = 0.4	756	0,09	No	Yes
39	2	M	Impaired cellular immunity	AD	From infancy	Detected<br>Birch pollen = 0.16	671	0,15	No	No
40	12	M	IgG subclass deficiency	A	From early childhood	Detected<br>Dog = 0.29	1304	0,28	No	No
41	12	F	IgG deficiency	A	From early childhood	Detected<br>BSA = 0.29	981	0,54	No	Yes

Abbreviations: * not detected - <0.15 kUa/L, ** detected – 0.15 kUa/L<sIgE<0.35 kUa/L; ***positive ≥0.35 kUa/L
AC – allergic conjunctivitis; AD – atopic dermatitis; AR – allergic rhinitis; A – asthma; BSA – bovine serum albumin; DIA – drug-induced anaphylaxis; F – female; FA – food allergy; IVIG (+) – immunoglobulin replacement therapy; M – male; No – number; PID – primary immunodeficiencies; PRKDC - Protein Kinase, DNA-Activated, Catalytic Subunit; U – urticaria.
