# Supplementary material for: Serum Allergen-Specific IgE among Pediatric Patients with Primary Immunodeficiency
Source: Children (Basel). 2022 Mar 25;9(4):466. doi: 10.3390/children9040466 (PMC9029572; doi:10.3390/children9040466)
Supplement: Supplementary file 1 [file children-09-00466-s001.zip › Children Table S1 9.pdf]

Table S1. Patients' baseline characteristics according to PID classification.

Type of PID	No of patients	No of men	No of women	Median age	Mean age	Immunoglobulin replacement therapy	HSCT
SCID	n=2 (2.78% of all)	n=2 (100.00%)	n=0 (0%)	4	4	n=2 (100.00%)	Before = n=1 (50.00%) After = n=1 (50.00%)
Ataxia-telangiectasia	n=4 (5.56% of all)	n=2 (50.00%)	n=2 (50.00%)	10	10	n=3 (75.00%)	n=0 (0%)
Nijmegen syndrome	n=3 (4.17% of all)	n=1 (33.33%)	n=2 (66.67%)	4	6	n=3 (100.00%)	n=0 (0%)
DiGeorge syndrome	n=2 (2.78% of all)	n=1 (50.00%)	n=1 (50.00%)	6	6	n=0 (0%)	n=0 (0%)
Kabuki syndrome	n=1 (1.39% of all)	n=1 (100.00%)	n=0 (0%)	14	14	n=1 (100.00%)	n=0 (0%)
PRKDC mutation associated with immunodeficiency	n=1 (1.39% of all)	n=1 (100.00%)	n=0 (0%)	9	9	n=1 (100.00%)	n=0 (0%)
Predominantly Ab deficiency (n=51):	n=51 (70.83% of all)	n= 35 (68.63%)	n=16 (31.37%)	7	8	n=9 (17.65%)	n=0 (0%)
- CVID (n=3)	n=3 (4.17% of all)	n=3 (100.00%)	n=0 (0%)	14	13	n=3 (100.00%)	
- X-linked agammaglobulinemia (n=1)	n=1 (1.39% of all)	n=1 (100.00%)	n=0 (0%)	15	15	n=1 (100.00%)	
- other hypo-gammaglobulinemia's* (n=20)	n= 20 (27.78% of all)	n= 15 (75.00%)	n= 5 (25.00%)	7	8	n=4 (20.00%)	
- IgG subclass deficiency (n=20)	n= 20 (27.78% of all)	n= 10 (50.00%)	n= 10 (50.00%)	7	7	n=1 (5%)	

- selective IgA deficiency (n=7)	n= 7 (9.72% of all)	n=6 (85.71%)	n=1 (14.29%)	7	9	n=0 (0%)	
Congenital defects of phagocyte number, function or both (n=3)	n= 3 (4.16% of all)	n=2 (66.67%)	n=1 (33.33%)	3	3	0% (n=0)	n=0 (0%)
Complement deficiency (n=2)	n=2 (2.77% of all)	n=1 (50.00%)	n=1 (50.00%)	5	5	n=0 (0%)	n=0 (0%)
Others (n=3)**	n=3 (4.16% of all)	n=1 (33.33%)	n=2 (66.67%)	7	7	n=0 (0%)	n=0 (0%)

*Other hypogammaglobulinemia's: IgG subclass deficiency with IgA deficiency/ IgG deficiency/ IgG and IgA deficiency/ IgM deficiency/ IgM and IgG subclass deficiency/ IgM and IgA deficiency/ IgM, IgG and IgA deficiency/ transient hypogammaglobulinemia of infancy.

Others**: congenital asplenia, lymphocyte T deficiency

Abbreviations: Ab – antibody; CVID – common variable immunodeficiency; HSCT – human stem cell transplantation; Ig – immunoglobulin; no-number; PRKDC – Protein Kinase, DNA-Activated, Catalytic Subunit; SCID – severe combined immunodeficiency
