# Supplementary material for: Serum Allergen-Specific IgE among Pediatric Patients with Primary Immunodeficiency
Source: Children (Basel). 2022 Mar 25;9(4):466. doi: 10.3390/children9040466 (PMC9029572; doi:10.3390/children9040466)
Supplement: Supplementary file 1 [file children-09-00466-s001.zip › Children Table S2 10.pdf]

Table S2. Characteristics of patients during immunoglobulin replacement therapy (n=19).

Patient No	Age	Gender	PID type	sIgE [kU/l]	IgE [IU/ml]	IgG [mg/dL]	Ig dose [g/kg]
1	15	M	X-linked agammaglobulinemia	BSA = 0.43	15.5	872 [518-1284]	0.3
2	14	M	CVID	BSA = 0.42	8.1	886 [518-1284]	0.45
3	15	M	CVID	<0.15	8.7	425 [518-1284]	0.4
4	12	M	CVID	BSA = 0.4	9.2	858 [518-1284]	0.3
5	9	M	IgG deficiency	BSA = 0.51	203.2	1145 [518-1284]	0.3
6	4	M	IgG deficiency	<0.15	9.1	849 [468-1150]	0.25
7	12	F	IgG deficiency	BSA = 0.27	47	981 [553-1631]	0.3
8	8	F	IgG subclass deficiency	<0.15	8.3	742 [553-1232]	0.3
9	7	M	IgG subclass and IgM deficiency	D.pteronyssinus >100<br>D. farinae >100<br>Cat = 1.2<br>Dog = 2.2	328.6	826 [553-1232]	0.35
10	9	M	PRKDC mutation<br>IgG subclass deficiency	6 Grass mix =4.1<br>Birch pollen =0.36<br>Mugwort pollen = 0.88<br>Cat = 0.96<br>Dog = 0.63	131	969 [518-1284]	0.35
11	14	M	Kabuki syndrome<br>IgG subclass deficiency	BSA = 0.27	2.3	1060 [518-1284]	0.25
12	4	M	Nijmegen breakage syndrome	BSA = 1.8	10	756 [468-1150]	0.5

13	3	F	Nijmegen breakage syndrome	BSA = 0.18	8.5	929 [468-1150]	0.5
14	11	F	Nijmegen breakage syndrome	6 Grass mix = 0.2 Birch pollen =0.18 Cat = 0.2 Dog = 0.2 BSA = 0.17	<10	663 [553-1631]	0.2
15	10	M	Ataxia-telangiectasia	Cow's milk = 0.16 BSA = 0.41	<1,5	1018 [518-1284]	0.3
16	11	K	Ataxia-telangiectasia	BSA = 0.67	9	906 [553-1631]	0.3
17	11	M	Ataxia-telangiectasia	BSA = 0.45	8.6	815 [540-1822}	0.5
18	2	M	SCID	<0.15	22.6	1016 [408-1120]	0.3
19	5	M	SCID	BSA = 0.37	7.5	898 [468-1150]	0.45

Abbreviations: BSA - bovine serum albumin; CVID – common variable immunodeficiency; Ig – immunoglobulin; No – number; PID – primary immunodeficiency; PRKDC - Protein Kinase, DNA-Activated, Catalytic Subunit; SCID – severe combined immunodeficiency; sIgE – specific IgE
